# Associations of milk, dairy products, calcium and vitamin D intake with risk of developing Parkinson´s disease within the EPIC4ND cohort

**DOI:** 10.1007/s10654-024-01183-9

**Published:** 2024-12-03

**Authors:** Mareike Gröninger, Jara Sabin, Rudolf Kaaks, Pilar Amiano, Dagfinn Aune, Natalia Cabrera Castro, Marcela Guevara, Johnni Hansen, Jan Homann, Giovanna Masala, Geneviève Nicolas, Susan Peters, Carlotta Sacerdote, Maria-Jose Sánchez, Maria Santucci De Magistris, Sabina Sieri, Roel Vermeulen, Yujia Zhao, Christina M. Lill, Verena A. Katzke

**Affiliations:** 1 Division of Cancer Epidemiology, German Cancer Research Center, Heidelberg, Germany; 2 University Clinics Heidelberg-Mannheim, Mannheim, Germany; 3 Ministry of Health of the Basque Government, Sub Directorate for Public Health and Addictions of Gipuzkoa, San Sebastián, Spain; 4 Biogipuzkoa (BioDonostia) Health Research Institute, Epidemiology of Chronic and Communicable Diseases Group, San Sebastián, Spain; 5 Department of Epidemiology and Biostatistics, School of Public Health, Imperial College London, London, UK; 6 Department of Research, Cancer Registry of Norway, Norwegian Institute of Public Health, Oslo, Norway; 7 Department of Nutrition, Oslo New University College, Oslo, Norway; 8 Department of Epidemiology, Regional Health Council, IMIB-Arrixaca, Murcia, Spain; 9 CIBER Epidemiología y Salud Pública (CIBERESP), Madrid, Spain; 10 Instituto de Salud Pública y Laboral de Navarra, Pamplona, 31003 Spain; 11 Centro de Investigación Biomédica en Red de Epidemiología y Salud Pública (CIBERESP), Madrid, 28029 Spain; 12 Navarra Institute for Health Research (IdiSNA), Pamplona, 31008 Spain; 13 Danish Cancer Institute, Danish Cancer Society, Copenhagen, Denmark; 14 Institute of Epidemiology and Social Medicine, University of Münster, Münster, Germany; 15 Clinical Epidemiology Unit, Institute for Cancer Research, Prevention and Clinical Network (ISPRO), Florence, Italy; 16 International Agency for Research on Cancer, Lyon, France; 17 Institute for Risk Assessment Sciences, Utrecht University, Utrecht, The Netherlands; 18 Department of Health Sciences, University of Eastern Piedmont, Novara, Italy; 19 Escuela Andaluza de Salud Pública (EASP), Granada, 18011 Spain; 20 Instituto de Investigación Biosanitaria ibs.GRANADA, Granada, 18012 Spain; 21 A.O.U. Federico II, Naples, Italy; 22 Fondazione IRCCS Istituto Nazionale dei Tumori, Milan, Italy; 23 University Medical Centre Utrecht, Utrecht, The Netherlands; 24 Ageing Epidemiology Research Unit, School of Public Health, Imperial College London, London, UK

**Keywords:** Dairy, Calcium, Vitamin D, Parkinson´s disease, EPIC

## Abstract

Literature indicates a potential association between dairy consumption and risk of Parkinson´s disease (PD), especially among men, yet the results remain inconclusive. We investigated this association in a large prospective European cohort. Dietary and non-dietary data was collected from 183,225 participants of the EPIC-for-Neurodegenerative-Diseases (EPIC4ND) cohort, a sub-cohort of the European Prospective Investigation into Cancer and Nutrition (EPIC) cohort. Crude and multivariable-adjusted Cox proportional hazards models were employed to examine potential associations between baseline dietary intake of dairy, calcium and vitamin D with incident PD risk. No relationship was observed between dairy consumption (HR 1.07, 95% CI 0.82–1.39), individual dairy products (milk: HR 0.95, 95% CI 0.73–1.23; yogurt: HR 1.03, 95% CI 0.82–1.29; cheese: HR 1.13, 95% CI 0.85–1.51), or vitamin D (HR 1.08, 95% CI 0.80–1.45) with PD risk. However, we observed a risk-increasing association with higher calcium intakes (HR 1.33, 95% CI 1.00-1.78, *p* for trend = 0.031), which was more pronounced in men (HR 1.50, 95% CI 1.00-2.25, *p* for trend = 0.044) and in ever smokers (HR 1.64, 95% CI 1.06–2.53, *p* for trend = 0.014). No compelling evidence was found for an association between dairy products or vitamin D intake and PD risk indicating a potentially limited relevance of dairy intake in PD risk than previously described. Our observations of a positive association between dietary calcium intake and PD risk in men and in ever smokers require further validation.

**Supplementary Information:**

The online version contains supplementary material available at 10.1007/s10654-024-01183-9.

## Introduction

Parkinson´s disease (PD) is the second most common neurodegenerative disorder [1], affecting approximately 8.5 million individuals globally in 2019 [2]. This represents a significant increase compared to 1990, with the global prevalent number increasing by 155% from 1990 [2]. Age-standardized prevalence was 1.4 times greater in men than in women in 2016. A rapid rise in prevalence is further expected as longevity increases and disease duration prolongs [3]. Although a rise in incidence is not definitely established, it is particularly hypothesized to be more pronounced among men owing to environmental risk factors [4].

The increasing number of PD cases, coupled with augmenting costs to health care systems and the patients´ impaired quality of life, underscore the need for research on modifiable risk factors for PD. It is well established that, beyond age and genetics, environmental, lifestyle, and medical factors interact to increase the risk of PD [5]. Specifically, established modifiable risk factors comprise pesticide exposure and prior head injury [1, 4]. In contrast, tobacco smoking, coffee consumption, higher serum urate concentrations, and physical activity have been associated with a decreased risk for PD [1, 4, 6, 7]. Several additional risk and protective factors have been reported but with contradicting results [4]. However, cause-effect relationships often remain unclear, possibly due in part to the scarcity of large, prospective cohorts that have been followed longitudinally over extended periods of time.

Dairy products are an important source of vitamins and minerals, such as calcium and vitamin D, and may reduce the risk of colorectal cancer [8, 9]. However, several studies indicate a positive association between dairy food intake and PD risk [10–12]. Furthermore, a pair of studies have observed an increased risk for PD in individuals with vitamin D insufficiency or deficiency [13, 14]. Further, a large nested case-control study from Denmark showed a decreased risk of PD in outdoor workers. This group is likely to have higher levels of vitamin D due to increased exposure to UV radiation, which is by far the most important source of vitamin D [15]. With the rise in number of PD cases and the widespread consumption of dairy products, often promoted for their high protein and calcium content, as well as the popularity of vitamin D supplementation for disease prevention, the question of a potential association between dairy products, calcium and vitamin D intake and PD risk emerges. As existing epidemiological evidence on this subject has primarily been derived from retrospective case-control and from non-European cohort studies, there is a need for large-scale epidemiological investigations within European cohorts to assess the association between dairy, calcium and vitamin D intake and the development of PD.

Therefore, we investigated the associations of milk, dairy products, calcium and vitamin D intake with the risk of developing PD within the EPIC-for-Neurodegenerative-Diseases (EPIC4ND) cohort – a large subset of the multicenter European Prospective Investigation into Cancer and Nutrition (EPIC) cohort.

## Materials and methods

### Study population

The EPIC study is a large multicenter cohort study including 521,323 participants enrolled between 1992 and 2000 in 23 centers across 10 European countries. EPIC aims to investigate the relationship between nutrition and lifestyle with cancer. At baseline, data on dietary and non-dietary variables, anthropometric measurements, and blood samples were collected from participants residing in the surrounding area of the given study center. Written informed consent was obtained from each participant, and the EPIC study was ethically approved by IARC and the respective centers [16, 17].

### EPIC4ND

**Fig. 1:**
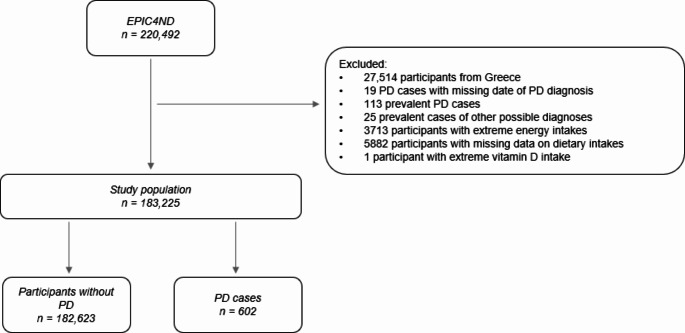
Flowchart of cohort participants included in the analysis

The EPIC4ND cohort, which is nested within the EPIC cohort, aims to investigate the association between pre-diagnostic risk factors and the development of PD, amongst other neurodegenerative disorders. It comprises data from 220,492 subjects in 13 of the 23 EPIC centers across 7 out of 10 European EPIC countries. For the present study, we used data from 183,225 subjects recruited in Sweden (Umeå, Malmö), the UK (Cambridge), the Netherlands (Utrecht), Germany (Heidelberg), Spain (Navarra, San Sebastian, Murcia) and Italy (Turin, Varese, Florence, Naples). The Naples and Utrecht cohorts only include women contrary to the remaining centers [17]. Figure 1 depicts a flowchart of the inclusion criteria for the study population. 27,514 participants from Greece were excluded due to GDPR issues. Furthermore, 19 PD cases with a missing date of diagnosis, 113 prevalent PD cases, defined as having received a diagnosis of PD before the date of recruitment or the same day, and 25 prevalent cases of other possible diagnoses, identified after case ascertainment and distinct to Parkinson´s disease, were also excluded. 3713 participants whose energy intake/energy requirement ratios fell outside the top or bottom 1% of all participants were excluded, alongside 5882 participants with missing data on dietary intakes. In addition, one participant exhibiting a vitamin D intake twice as high as the second highest intake level was also excluded due to implausibility.

### Dietary intake assessments

Data on dietary intake was assessed at baseline using different dietary assessment methods across countries and centers. In Italy, the UK, the Netherlands, Sweden and Germany extensive (semi-quantitative) food-frequency questionnaires (FFQs) were used to estimate individual average portion size. Questionnaires were self-administered, except for Naples in Italy, were face-to-face semi-quantitative FFQs were conducted. In Spain, face-to-face diet history questionnaires, structured by meals, were administered. In Malmö, in addition to the FFQ, data was collected through a 7-day record on hot meals [18]. 

To address the issue of limited comparability due to different dietary assessment methods, additional data on dietary intake was obtained through a computer-assisted 24-hour dietary recall (24-HDR) in representative sub-samples. These sub-samples encompassed 5–12% of study participants in each sub-cohort, except for the UK (1.5%). Data from the 24-HDR served as a reference method to address systematic over- or underestimation between centers in the initial dietary assessments [19]. 

Values for daily energy intake, calcium and vitamin D intakes were computed based on country-specific food composition tables, which were harmonized across the countries participating in EPIC (EPIC Nutrient DataBase, ENDB) [20].

### Non-dietary variables

Information on lifestyle, menstrual and reproductive history, and past medical history was collected through questions on education, socio-economic status, and occupation; history of previous illness, disorders or surgical operations; lifetime history of tobacco use; lifetime history of alcohol consumption; physical activity; menstrual and reproductive history; and utilization of hormones for contraception and postmenopausal replacement therapy. Height, weight, and waist and hip circumference were measured on all subjects in all centers at baseline, except for Umeå where only weight and height were measured [16]. 

### Outcome assessment

For ascertainment of PD cases, an EPIC4ND template for clinical data collection was developed upon which a possible final diagnosis was made. These included PD, multiple system atrophy, progressive supranuclear palsy, vascular parkinsonism, dementia with Lewy bodies, essential tremor, PD with essential tremor and unclassifiable parkinsonism, amongst others. Depending on the amount and quality of available data (“poor”, “good” or “excellent”) as well as the extent of confidence of the neurologist expert (“low”, “medium” and “high”), each diagnosis was thereafter labelled with an EPIC4ND label (“definite”, “very likely”, “probable” or “possible”). Subsequently, ascertainment consisted of two phases: In phase I, potential cases were identified via record linkage with at least one local source of information to minimize the chances of false negatives. Resources for record linkage encompass hospital discharge registries, drug databases, mortality records, questionnaires, amongst others. In phase II, said potential cases were re-reviewed by specialists in movement disorders and a final diagnosis was established according to the standardized protocol described above and previously [17]. 

### Statistical analysis

Descriptive statistics were calculated separately for participants that remained without PD diagnosis during follow-up, and for incident PD patients, with median and interquartile range for continuous variables, and proportions for categorical variables. Crude, basic- (sex-, age- and country-adjusted) and multivariable-adjusted hazard ratios (HRs) and their 95% Confidence Intervals (CIs) were estimated from the Cox proportional hazards model with age as the underlying time scale for each exposure of interest: total dairy, milk, yogurt, cheese, calcium and vitamin D intake. Dietary exposure variables under study were classified into sex-specific quartiles based on the distribution of intake observed among non-cases. Separate models were fit for each exposure of interest, using the lowest quartile as the reference category. *P* for trend was calculated by entering the quartile indicator variable into the models as a continuous variable. Additionally, HRs and 95% CIs were estimated from Cox proportional hazards model for every additional 100 g/d consumption of milk separately by fat content (skimmed, semi-skimmed and whole milk).

Male gender, age, smoking and coffee consumption are widely acknowledged as factors related to PD risk [1, 6, 7] and they may influence dietary and dairy consumption habits. Therefore, the multivariable-adjusted analysis was adjusted for these factors. Furthermore, we included previously established confounders into the multivariable-adjusted model, such as BMI, highest level of school education, physical activity, alcohol consumption and diabetes status. Since this study aims to investigate the risk of PD in association with dietary factors, we further adjusted for energy intake. Additionally, adjustment was made for country due to differences in data collection methods across study centers and variations in dietary habits across EPIC countries. In conclusion, the main model was adjusted for sex, age at recruitment, country of residence, BMI, highest education level (none, primary school completed, technical/professional school, secondary school, longer education, or not specified), physical activity (inactive, moderately inactive, moderately active, active, or not specified), smoking history (never, current (1–15 cig/day), current (16–25 cig/day), current (26 + cig/day), former (quit ≤ 10 years), former (quit 11–20 years), former (quit 20 + years), current (pipe/cigar/occas), or not specified), alcohol consumption, coffee consumption, diabetes status (no, yes, or not specified) and energy intake. BMI (kg/m^2^), alcohol consumption (g/day), coffee consumption (ml/day) and energy intake (kcal/d) were included in the model as continuous variables. To account for proportional hazards, we adjusted for age in 5-year steps and stratified the analysis by country. In a supplementary analysis, we investigated the associations of the individual covariates with PD risk.

Furthermore, we conducted sensitivity analyses by excluding the first 5 years of follow-up to reduce potential reverse causation bias. In a second sensitivity analysis, we only included cases with a PD diagnosis that had previously been labelled as “definite” or “very likely” (*n* = 314).

Since female gender and smoking confer as potential protective factors for PD, and conflicting evidence concerning the influence of overweight exists, we evaluated potential effect modification by running the analyses stratified by sex, by smoking status (never and ever smokers) and by BMI (BMI < 25 and BMI ≥ 25). Due to differences in dietary assessment methods across country, we conducted separate analyses for each country. For the latter, we modelled the dietary variables continuously, as there were too few cases for subgroup analysis by quartiles. Statistical heterogeneity between subgroups was assessed using interaction terms and the Wald statistic.

Additionally, we investigated the association of calcium and vitamin D with PD risk by food sources (from dairy products and from other food sources) overall, in men and in women. Consumption of calcium and vitamin D from other sources was computed by calculating calcium_*total*_ – calcium_*dairy*_ or vitamin D_*total*_ – vitamin D_*dairy*_. Sex-specific quartiles were calculated individually for calcium intake from dairy, calcium intake from other sources, vitamin D intake from dairy and vitamin D intake from other sources among non-PD-diseased participants.

Statistical significance was set at the α = 0.05 level. The data analysis for this paper was generated using R software version 4.3.1 [21], and SAS software, Version 9.4. Copyright © 2024 SAS Institute Inc. SAS and all other SAS Institute Inc. product or service names are registered trademarks or trademarks of SAS Institute Inc., Cary, NC, USA.

## Results

### Baseline characteristics

Table 1 displays participant characteristics of the study cohort by status of incident PD diagnosis. During a median follow-up time of 17.59 years, 602 incident PD cases were identified, of which 117 diagnoses were labelled as ‘definite’, 197 as ‘very likely’, 116 as ‘probable’ and 172 as ‘possible’. Median time from baseline until PD diagnosis was 8.3 years (IQR 4.9–11.7) with a median age at diagnosis of 70 years. The majority of the study population was female (63%), whereas incident PD cases were distributed almost evenly across gender (48% female vs. 52% male). Associations of the individual covariates with PD risk are depicted in supplementary Table 1 (see Online Resource).

**Table 1:** Selected baseline characteristics of *N* = 183,225 participants of the EPIC4ND study by Parkinson´s disease diagnosis status

Variable	Parkinson’s Disease	
	No^a^, *N* = 182,623	Yes^a^, *N* = 602
**Age at recruitment**		
Median (IQR)	53 (47–60)	61 (55–66)
Range	24–79	30–76
**Age at PD diagnosis**		
Median (IQR)	NA	70 (64–74)
**Sex**		
Male	67,534 (37%)	311 (52%)
Female	115,089 (63%)	291 (48%)
**Country**		
Italy	39,578 (22%)	64 (11%)
Spain	24,418 (13%)	96 (16%)
United Kingdom	24,593 (13%)	187 (31%)
The Netherlands	16,563 (9%)	13 (2%)
Germany	24,965 (14%)	48 (8%)
Sweden	52,506 (29%)	194 (32%)
**BMI** (kg/m^2^)		
Low BMI (< 18.5)	1,766 (1%)	6 (1%)
Normal BMI (≥ 18.5 - ≤ 24.9)	78,970 (43%)	224 (37%)
High BMI (> 24.9)	101,887 (56%)	372 (62%)
**Highest school education**		
None	8,679 (5%)	49 (8%)
Primary school completed	66,708 (37%)	237 (39%)
Technical/Professional school	42,402 (23%)	142 (24%)
Secondary school	29,212 (16%)	63 (10%)
Longer education (incl. University deg.)	32,327 (18%)	84 (14%)
Unknown	3,295 (2%)	27 (4%)
**Physical activity**		
Inactive	24,507 (13%)	64 (11%)
Moderately inactive	45,630 (25%)	182 (30%)
Moderately active	69,718 (38%)	259 (43%)
Active	15,778 (9%)	32 (5%)
Unknown	26,990 (15%)	65 (11%)
**Smoking status and intensity of smoking**		
Never	76,764 (42%)	305 (51%)
Current, 1–15 cig/day	22,349 (12%)	38 (6%)
Current, 16–25 cig/day	11,831 (6%)	17 (3%)
Current, 26 + cig/day	3,248 (2%)	2 (0%)
Former, quit < = 10 years	18,393 (10%)	50 (8%)
Former, quit 11–20 years	16,726 (9%)	39 (6%)
Former, quit 20 + years	15,716 (9%)	96 (16%)
Current, pipe/cigar/occas	12,513 (7%)	32 (5%)
Unknown	5,083 (3%)	23 (4%)
**Alcohol consumption** (g/d)		
Non drinker	28,972 (16%)	113 (19%)
>0–6(M)/>0–3(W)	56,100 (31%)	173 (29%)
>6–12(M)/>3–12(W)	41,610 (23%)	140 (23%)
>12–24	27,164 (15%)	93 (15%)
>24–60	24,328 (13%)	73 (12%)
>60	4,449 (2%)	10 (2%)
**Coffee consumption**		
No coffee	12,780 (7%)	68 (11%)
Up to 1 cup	57,147 (31%)	156 (26%)
1–3 cups	56,990 (31%)	207 (34%)
3–5 cups	39,521 (22%)	126 (21%)
More than 5 cups	16,185 (9%)	45 (7%)
**Diabetes mellitus**		
No	150,462 (82%)	376 (62%)
Yes	5,038 (3%)	32 (5%)
Unknown	27,123 (15%)	194 (32%)
**Energy intake** (kcal/d)		
Median (IQR)	2,009 (1,634-2,464)	2,094 (1,699-2,522)
**Dairy consumption** (g/d)		
Median (IQR)	300 (175–464)	345 (203–529)
Range	0–4,566	0–1,176
**Milk consumption** (g/d)		
Median (IQR)	164 (49–300)	239 (80–438)
Range	0–3,015	0–1,006
**Yogurt consumption** (g/d)		
Median (IQR)	30 (0–99)	18 (0–99)
Range	0–3,165	0–575
**Cheese consumption** (g/d)		
Median (IQR)	29 (15–50)	22 (13–42)
Range	0–561	0–260
**Calcium intake** (mg/d)		
Median (IQR)	950 (731-1,211)	1,010 (771-1,275)
Range	111–8,893	164–2,372
**Vitamin D intake** (µg/d)		
Median (IQR)	4 (2–6)	4 (3–6)
Range	0–65	1–19

^a^ Median (IQR) or Frequency (%)

### Associations of dairy products with PD risk

Associations between dietary intake and PD risk were assessed using Cox proportional hazards model. Total dairy consumption and milk alone were positively associated with an increased PD risk in crude models, but not after basic or multivariable adjustment (Table 2). The multivariable-adjusted HR comparing the highest versus lowest quartile of intake was 1.07 (95% CI 0.82–1.39, *p* for trend = 0.6) for total dairy and 0.95 (95% CI 0.73–1.23, *p* for trend > 0.9) for milk. When examining associations for milk separately by fat content, PD risk increased by 5% for every additional 100 g/day increment of semi-skimmed milk (HR 1.05, 95% CI 1.00-1.10) (supplementary Table 2). No significant associations between total dairy or milk intake with PD risk were observed in multivariable-adjusted models in subgroup analyses of sex, smoking status and BMI (Table 3, supplementary Tables 3 and 4), whereas a significant positive association was observed among participants from the UK for every additional 200 g of dairy (HR 1.21, 95% CI 1.02–1.43) and milk intake (HR 1.20, 95% CI 1.00-1.44) (supplementary Table 5).

**Table 2:** Analysis of the association between quartiles of dietary intake of total dairy, milk, yogurt, cheese, calcium and vitamin D, and incident Parkinson´s disease in the EPIC4ND cohort: crude, basic- and multivariable-adjusted Hazard Ratios (HRs) and 95% confidence intervals (CIs) obtained from Cox proportional hazards model

Variable	Quartile				*P* for trend
	1 (lowest)	2	3	4 (highest)	
**Total dairy** (g/day)^a^ (men/women)	≤ 156.19/≤ 184.95	≤ 294.29/≤ 302.69	≤ 476.02/≤ 456.96	> 476.02/> 456.96	
Event N	118	125	162	197	
Crude HR (95% CI)	Ref.	1.05 (0.82–1.35)	**1.37 (1.08–1.74)**	**1.67 (1.33–2.09)**	**< 0.001**
Basic-adj. HR (95% CI)^b^	Ref.	0.94 (0.73–1.22)	0.91 (0.71–1.17)	1.14 (0.89–1.46)	0.2
Multivariable HR (95% CI)^c^	Ref.	0.91 (0.71–1.17)	0.87 (0.67–1.12)	1.07 (0.82–1.39)	0.6
**Milk Intake** (g/day)^a^ (men/women)	≤ 36.21/≤ 56.40	≤ 164.29/≤ 163.91	≤ 312.76/≤ 296.22	> 312.76/> 296.22	
Event N	118	117	171	196	
Crude HR (95% CI)	Ref.	0.99 (0.76–1.27)	**1.44 (1.14–1.82)**	**1.67 (1.33–2.09)**	**< 0.001**
Basic-adj. HR (95% CI)^b^	Ref.	0.81 (0.63–1.06)	0.91 (0.71–1.18)	0.99 (0.77–1.27)	0.6
Multivariable HR (95% CI)^c^	Ref.	0.80 (0.62–1.04)	0.89 (0.69–1.15)	0.95 (0.73–1.23)	> 0.9
**Yogurt Intake** (g/day)^a^ (men/women)	0/≤ 1.19	≤ 17.86/≤ 35.71	≤ 89.29/≤ 100.00	> 89.29/> 100.00	
Event N	223	115	116	148	
Crude HR (95% CI)	Ref.	**0.68 (0.54–0.85)**	**0.61 (0.49–0.76)**	**0.76 (0.62–0.93)**	**0.002**
Basic-adj. HR (95% CI)^b^	Ref.	1.14 (0.90–1.45)	0.88 (0.70–1.11)	1.08 (0.87–1.35)	> 0.9
Multivariable HR (95% CI)^c^	Ref.	1.13 (0.89–1.43)	0.86 (0.68–1.08)	1.03 (0.82–1.29)	0.7
**Cheese Intake** (g/day)^a^ (men/women)	≤ 13.71/≤ 15.33	≤ 25.71/≤ 30.00	≤ 47.74/≤ 51.61	> 47.74/> 51.61	
Event N	177	159	156	110	
Crude HR (95% CI)	Ref.	0.90 (0.73–1.12)	0.88 (0.71–1.10)	**0.63 (0.49–0.79)**	**< 0.001**
Basic-adj. HR (95% CI)^b^	Ref.	1.08 (0.87–1.34)	**1.32 (1.05–1.66)**	1.19 (0.91–1.56)	0.053
Multivariable HR (95% CI)^c^	Ref.	1.06 (0.85–1.32)	**1.28 (1.01–1.61)**	1.13 (0.85–1.51)	0.14
**Calcium Intake** (mg/day)^a^ (men/women)	≤ 734.70/≤ 728.75	≤ 970.56/≤ 939.80	≤ 1249.38/≤ 1189.82	> 1249.38/> 1189.82	
Event N	124	137	164	177	
Crude HR (95% CI)	Ref.	1.11 (0.87–1.41)	**1.33 (1.05–1.68)**	**1.43 (1.14–1.80)**	**< 0.001**
Basic-adj. HR (95% CI)^b^	Ref.	1.01 (0.79–1.29)	1.14 (0.90–1.45)	**1.32 (1.04–1.67)**	**0.009**
Multivariable HR (95% CI)^c^	Ref.	1.00 (0.78–1.29)	1.14 (0.88–1.47)	1.33 (1.00–1.78)	**0.031**
**Vitamin D Intake** (µg/day)^a^ (men/women)	≤ 2.87/≤ 2.21	≤ 4.46/≤ 3.25	≤ 6.83/≤ 4.83	> 6.83/> 4.83	
Event N	120	135	156	191	
Crude HR (95% CI)	Ref.	1.12 (0.88–1.44)	**1.29 (1.02–1.64)**	**1.57 (1.25–1.98)**	**< 0.001**
Basic-adj. HR (95% CI)^b^	Ref.	1.08 (0.84–1.39)	1.09 (0.85–1.40)	1.13 (0.87–1.47)	0.4
Multivariable HR (95% CI)^c^	Ref.	1.06 (0.83–1.37)	1.06 (0.81–1.38)	1.08 (0.80–1.45)	0.7

^a^ Quartiles based on baseline dietary intake among non-cases

^b^ Adjusted for sex, age, country

^c^ Adjusted for sex, age, country, body mass index, education, physical activity, smoking history, alcohol intake, coffee consumption, diabetes, energy intake

**Table 3:** Analysis of the association between quartiles of dietary intake of total dairy, milk, yogurt, cheese, calcium and vitamin D, and incident Parkinson´s disease **stratified by sex** in the EPIC4ND cohort: crude and multivariable-adjusted Hazard Ratios (HRs) and 95% confidence intervals (CIs) obtained from Cox proportional hazards model

Variable	Quartile				*P* for trend	*P*_het_
	1 (lowest)	2	3	4 (highest)		
**Total dairy**						
**Men** (g/day)^a^	≤ 156.19	≤ 294.29	≤ 476.02	> 476.02		
Event N	57	62	85	107		
Crude HR (95% CI)	Ref.	1.08 (0.76 – 1.55)	**1.50 (1.07–2.10)**	**1.89 (1.37–2.61)**	**< 0.001**	
Multivariable HR (95% CI)^b^	Ref.	0.94 (0.65–1.35)	0.94 (0.65–1.37)	1.32 (0.90–1.95)	0.11	
**Women** (g/day)^a^	≤ 184.95	≤ 302.69	≤ 456.96	> 456.96		
Event N	61	63	77	90		
Crude HR (95% CI)	Ref.	1.02 (0.72–1.46)	1.25 (0.89–1.75)	**1.46 (1.05–2.02)**	**0.010**	
Multivariable HR (95% CI)^b^	Ref.	0.86 (0.60–1.23)	0.79 (0.55–1.12)	0.85 (0.59–1.22)	0.4	0.9
**Milk Intake**						
**Men** (g/day)^a^	≤ 36.21	≤ 164.29	≤ 312.76	> 312.76		
Event N	63	55	89	104		
Crude HR (95% CI)	Ref.	0.87 (0.60–1.25)	**1.42 (1.03–1.96)**	**1.67 (1.22–2.28)**	**< 0.001**	
Multivariable HR (95% CI)^b^	Ref.	0.71 (0.49–1.03)	0.86 (0.60–1.23)	0.94 (0.65–1.36)	0.8	
**Women** (g/day)^a^	≤ 56.40	≤ 163.91	≤ 296.22	> 296.22		
Event N	55	62	82	92		
Crude HR (95% CI)	Ref.	1.12 (0.78–1.61)	**1.47 (1.04–2.06)**	**1.66 (1.19–2.33)**	**< 0.001**	
Multivariable HR (95% CI)^b^	Ref.	0.91 (0.63–1.32)	0.92 (0.64–1.34)	0.95 (0.66–1.38)	0.9	0.6
**Yogurt Intake**						
**Men** (g/day)^a^	0	≤ 17.86	≤ 89.29	> 89.29		
Event N	139	41	59	72		
Crude HR (95% CI)	Ref.	**0.69 (0.49–0.98)**	**0.59 (0.44–0.80)**	**0.73 (0.55–0.97)**	**0.005**	
Multivariable HR (95% CI)^b^	Ref.	0.97 (0.68–1.40)	0.88 (0.64–1.21)	1.09 (0.80–1.50)	0.8	
**Women** (g/day)^a^	≤ 1.19	≤ 35.71	≤ 100.00	> 100.00		
Event N	84	74	57	76		
Crude HR (95% CI)	Ref.	0.86 (0.63–1.17)	**0.70 (0.50–0.98)**	0.90 (0.66–1.22)	0.3	
Multivariable HR (95% CI)^b^	Ref.	1.25 (0.90–1.73)	0.85 (0.60–1.20)	1.00 (0.71–1.39)	0.5	0.7
**Cheese Intake**						
**Men** (g/day)^a^	≤ 13.71	≤ 25.71	≤ 47.74	> 47.74		
Event N	91	86	78	56		
Crude HR (95% CI)	Ref.	0.95 (0.71–1.28)	0.86 (0.63–1.16)	**0.61 (0.44–0.85)**	**0.004**	
Multivariable HR (95% CI)^b^	Ref.	0.92 (0.68–1.25)	1.08 (0.78–1.49)	1.06 (0.72–1.57)	0.6	
**Women** (g/day)^a^	≤ 15.33	≤ 30.00	≤ 51.61	> 51.61		
Event N	86	73	78	54		
Crude HR (95% CI)	Ref.	0.85 (0.62–1.16)	0.91 (0.67–1.24)	**0.64 (0.46–0.90)**	**0.024**	
Multivariable HR (95% CI)^b^	Ref.	1.16 (0.84–1.60)	**1.47 (1.05–2.07)**	1.20 (0.79–1.82)	0.2	0.8
**Calcium Intake**						
**Men** (mg/day)^a^	≤ 734.70	≤ 970.56	≤ 1249.38	> 1249.38		
Event N	65	71	77	98		
Crude HR (95% CI)	Ref.	1.09 (0.78–1.53)	1.19 (0.86–1.66)	**1.51 (1.10–2.07)**	**0.008**	
Multivariable HR (95% CI)^b^	Ref.	0.97 (0.68–1.37)	1.02 (0.71–1.48)	**1.50 (1.00–2.25)**	**0.044**	
**Women** (mg/day)^a^	≤ 728.75	≤ 939.80	≤ 1189.82	> 1189.82		
Event N	59	66	87	79		
Crude HR (95% CI)	Ref.	1.12 (0.79–1.59)	**1.48 (1.07–2.07)**	1.35 (0.96–1.89)	**0.030**	
Multivariable HR (95% CI)^b^	Ref.	1.01 (0.71–1.46)	1.22 (0.84–1.76)	1.10 (0.72–1.69)	0.5	0.7
**Vitamin D Intake**						
**Men** (µg/day)^a^	≤ 2.87/≤ 2.21	≤ 4.46/≤ 3.25	≤ 6.83/≤ 4.83	> 6.83/> 4.83		
Event N	70	81	83	77		
Crude HR (95% CI)	Ref.	1.16 (0.84–1.60)	1.18 (0.86–1.63)	1.10 (0.79–1.52)	0.6	
Multivariable HR (95% CI)^b^	Ref.	1.11 (0.79–1.54)	1.08 (0.76–1.55)	1.05 (0.68–1.60)	0.8	
**Women** (µg/day)^a^	≤ 2.21	≤ 3.25	≤ 4.83	> 4.83		
Event N	50	54	73	114		
Crude HR (95% CI)	Ref.	1.08 (0.73–1.58)	**1.44 (1.01–2.06)**	**2.23 (1.60–3.12)**	**< 0.001**	
Multivariable HR (95% CI)^b^	Ref.	1.02 (0.68–1.51)	1.05 (0.71–1.57)	1.09 (0.70–1.69)	0.7	0.066

^a^ Quartiles based on baseline dietary intake among non-cases

^b^ Adjusted for age, country, body mass index, education, physical activity, smoking history, alcohol intake, coffee consumption, diabetes, energy intake

Considering the intake of the individual dairy items yogurt and cheese, significant risk-decreasing effect estimates observed for higher intake levels in univariate models were deemed non-significant in multivariable models (yogurt: *p* for trend = 0.7, cheese: *p* for trend = 0.14) (Table 2). In never smokers, the significant inverse association between yogurt intake and PD risk remained after multivariable adjustment with a HR comparing the highest vs. lowest quartile of intake of 0.71 (95% CI 0.52–0.98, *p* for trend = 0.014), whereas in ever smokers, we observed a significant positive association of yogurt intake and PD risk (multivariable HR 1.55, 95% CI 1.12–2.16, *p* for trend = 0.040, *p*_*het*_ = 0.2) (supplementary Table 3). Subgroup analyses of cheese intake yielded non-significant results after multivariable adjustment (Table 3, supplementary Tables 3 and 4).

### Associations of dietary calcium and vitamin D intake with PD risk

In the nutrient analyses, dietary calcium and vitamin D intakes were associated with a higher PD risk in crude models (calcium: HR 1.43, 95% CI 1.14–1.80, *p* for trend < 0.001); vitamin D: HR 1.57, 95% CI 1.25–1.98, *p* for trend < 0.001) (Table 2). Additionally, we observed a risk-increasing association for calcium in the age-, sex- and country-adjusted model (*p* for trend = 0.009) as well as after multivariable adjustment (*p* for trend = 0.031). In subgroup analysis, the observed association was only present among men (HR 1.50, 95% CI: 1.00-2.25, *p* for trend = 0.044, *p*_*het*_ = 0.7) (Table 3), among ever smokers (HR 1.64, 95% CI 1.06–2.53, *p* for trend = 0.014, *p*_*het*_ = 0.014) (supplementary Table 3), and among participants from the UK for every additional 200 mg of calcium intake per day (HR 1.17, 95% CI 1.03–1.32) (supplementary Table 5).

Regarding dietary vitamin D intakes, no association with PD risk was evident overall (*p* for trend = 0.7), or separately by sex, smoking status, BMI or country after multivariable adjustment (Tables 2 and 3, supplementary Tables 3–5).

In further analysis, we investigated the association between calcium and vitamin D from dairy and from other food sources with PD risk (Table 4). The previously observed risk-increasing association of calcium intake was evident in the multivariable-adjusted model of calcium from dairy products (*p* for trend = 0.047), but not for calcium derived from other foods (*p* for trend = 0.4). Additionally, we observed a risk-increasing association between vitamin D from dairy products with PD risk (multivariable-adjusted HR 1.45, 95% CI:1.07–1.96, *p* for trend = 0.014). No significant association with risk of developing PD was evident for vitamin D intake derived from non-dairy products. Associations found for nutrients from dairy products seem to be modified by sex such that dairy calcium is associated with PD in men (HR 1.49, 95% CI 1.03–2.16, *p* for trend = 0.024), and dairy vitamin D in women (HR 1.58, 95% CI 1.03–2.43, *p* for trend = 0.065) only. However, heterogeneity between sexes was only found to be significant for the association between vitamin D from dairy intake and PD risk (*p*_*het*_ = 0.049).

**Table 4:** Analysis of the association between quartiles of dietary intake of calcium and vitamin D **by food source** (dairy, non-dairy) and incident Parkinson´s disease in the EPIC4ND cohort: crude, basic- and multivariable-adjusted Hazard Ratios (HRs) and 95% confidence intervals (CIs) obtained from Cox proportional hazards model

Variable	Quartile				*P* for trend	*P*_het_
	1 (lowest)	2	3	4 (highest)		
**Calcium intake from dairy** (mg/day)^a^ (men/women)	≤ 384.33/≤ 389.61	≤ 556.34/≤ 572.12	≤ 814.02/≤ 797.80	> 814.02/> 797.80		
Event N	130	144	157	171		
Crude HR (95% CI)	Ref.	1.11 (0.88–1.41)	1.21 (0.96–1.53)	**1.32 (1.05–1.66)**	**0.012**	
Basic-adj. HR (95% CI)^b^	Ref.	0.97 (0.76–1.24)	1.04 (0.82–1.33)	**1.33 (1.05–1.69)**	**0.011**	
Multivariable HR (95% CI)^c^	Ref.	0.95 (0.74–1.21)	1.01 (0.79–1.29)	1.28 (0.99–1.65)	**0.047**	
**Calcium intake from dairy** (mg/day)^a^ **Men**	≤ 384.33	≤ 556.34	≤ 814.02	> 814.02		
Event N	65	74	79	93		
Crude HR (95% CI)	Ref.	1.14 (0.82–1.59)	1.22 (0.88–1.70)	**1.43 (1.04–1.97)**	**0.023**	
Basic-adj. HR (95% CI)^b^	Ref.	0.98 (0.69–1.38)	1.00 (0.71–1.42)	**1.44 (1.03–2.02)**	**0.025**	
Multivariable HR (95% CI)^c^	Ref.	0.96 (0.68–1.36)	1.02 (0.71–1.45)	**1.49 (1.03–2.16)**	**0.024**	
**Calcium intake from dairy** (mg/day)^a^ **Women**	≤ 389.61	≤ 572.12	≤ 797.80	> 797.80		
Event N	65	70	78	78		
Crude HR (95% CI)	Ref.	1.08 (0.77–1.51)	1.20 (0.87–1.67)	1.20 (0.87–1.67)	0.2	
Basic-adj. HR (95% CI)^b^	Ref.	0.97 (0.69–1.37)	1.08 (0.77–1.51)	1.24 (0.89–1.74)	0.2	
Multivariable HR (95% CI)^c^	Ref.	0.92 (0.66–1.30)	0.98 (0.69–1.38)	1.05 (0.73–1.52)	0.7	0.9
**Calcium intake from non-dairy** (mg/day)^a^ (men/women)	≤ 301.78/≤ 278.33	≤ 394.10/≤ 347.47	≤ 497.80/≤ 431.93	> 497.80/> 431.93		
Event N	124	139	154	185		
Crude HR (95% CI)	Ref.	1.12 (0.88–1.43)	1.24 (0.98–1.57)	**1.50 (1.20–1.88)**	**< 0.001**	
Basic-adj. HR (95% CI)^b^	Ref.	1.01 (0.79–1.30)	1.07 (0.84–1.37)	1.17 (0.91–1.50)	0.2	
Multivariable HR (95% CI)^c^	Ref.	1.00 (0.76–1.30)	1.05 (0.79–1.41)	1.14 (0.81–1.60)	0.4	
**Calcium intake from non-dairy** (mg/day)^a^ **Men**	≤ 301.78	≤ 394.10	≤ 497.80	> 497.80		
Event N	61	74	78	98		
Crude HR (95% CI)	Ref.	1.22 (0.87–1.71)	1.27 (0.91–1.78)	**1.60 (1.16–2.21)**	**0.004**	
Basic-adj. HR (95% CI)^b^	Ref.	0.95 (0.67–1.35)	0.93 (0.65–1.32)	1.06 (0.74–1.52)	0.7	
Multivariable HR (95% CI)^c^	Ref.	0.96 (0.66–1.41)	0.96 (0.63–1.46)	1.14 (0.70–1.86)	0.5	
**Calcium intake from non-dairy** (mg/day)^a^ **Women**	≤ 278.33	≤ 347.47	≤ 431.93	> 431.93		
Event N	63	65	76	87		
Crude HR (95% CI)	Ref.	1.04 (0.73–1.46)	1.21 (0.87–1.69)	**1.40 (1.01–1.94)**	**0.024**	
Basic-adj. HR (95% CI)^b^	Ref.	1.06 (0.74–1.50)	1.21 (0.86–1.71)	1.28 (0.90–1.83)	0.12	
Multivariable HR (95% CI)^c^	Ref.	0.99 (0.67–1.45)	1.07 (0.71–1.60)	1.01 (0.62–1.63)	0.9	0.6
**Vitamin D intake from dairy** (µg/day)^a^ (men/women)	≤ 0.107/≤ 0.116	≤ 0.229/≤ 0.217	≤ 0.573/≤ 0.432	> 0.573/> 0.432		
Event N	184	136	123	159		
Crude HR (95% CI)	Ref.	**0.74 (0.59–0.92)**	**0.66 (0.53–0.83)**	0.85 (0.69–1.05)	0.073	
Basic-adj. HR (95% CI)^b^	Ref.	1.11 (0.88–1.39)	1.28 (0.99–1.64)	**1.51 (1.13–2.02)**	**0.004**	
Multivariable HR (95% CI)^c^	Ref.	1.08 (0.86–1.37)	1.23 (0.95–1.59)	**1.45 (1.07–1.96)**	**0.014**	
**Vitamin D intake from dairy** (µg/day)^a^ **Men**	≤ 0.107	≤ 0.229	≤ 0.573	> 0.573		
Event N	111	64	70	66		
Crude HR (95% CI)	Ref.	**0.57 (0.42–0.78)**	**0.62 (0.46–0.84)**	**0.58 (0.43–0.79)**	**< 0.001**	
Basic-adj. HR (95% CI)^b^	Ref.	0.82 (0.60–1.13)	1.16 (0.84–1.61)	1.29 (0.85–1.96)	0.2	
Multivariable HR (95% CI)^c^	Ref.	0.82 (0.59–1.13)	1.14 (0.81–1.61)	1.27 (0.82–1.97)	0.2	
**Vitamin D intake from dairy** (µg/day)^a^ **Women**	≤ 0.116	≤ 0.217	≤ 0.432	> 0.432		
Event N	73	72	53	93		
Crude HR (95% CI)	Ref.	0.99 (0.72–1.38)	0.72 (0.51–1.03)	1.24 (0.91–1.69)	0.4	
Basic-adj. HR (95% CI)^b^	Ref.	**1.55 (1.10–2.17)**	1.43 (0.97–2.10)	**1.77 (1.17–2.67)**	**0.011**	
Multivariable HR (95% CI)^c^	Ref.	**1.46 (1.03–2.06)**	1.29 (0.87–1.93)	**1.58 (1.03–2.43)**	0.065	**0.049**
**Vitamin D intake from non-dairy** (µg/day)^a^ (men/women)	≤ 2.618/≤ 1.968	≤ 4.036/≤ 2.918	≤ 6.082/≤ 4.297	> 6.082/> 4.297		
Event N	120	134	152	196		
Crude HR (95% CI)	Ref.	1.12 (0.87–1.43)	1.26 (0.99–1.60)	**1.62 (1.29–2.03)**	**< 0.001**	
Basic-adj. HR (95% CI)^b^	Ref.	1.04 (0.81–1.34)	1.03 (0.80–1.32)	1.06 (0.83–1.37)	0.7	
Multivariable HR (95% CI)^c^	Ref.	1.03 (0.80–1.32)	0.99 (0.77–1.29)	1.00 (0.75–1.34)	> 0.9	
**Vitamin D intake from non-dairy** (µg/day)^a^ **Men**	≤ 2.618	≤ 4.036	≤ 6.082	> 6.082		
Event N	70	78	84	79		
Crude HR (95% CI)	Ref.	1.12 (0.81–1.54)	1.20 (0.87–1.65)	1.13 (0.82–1.56)	0.4	
Basic-adj. HR (95% CI)^b^	Ref.	1.05 (0.75–1.45)	1.05 (0.75–1.48)	0.94 (0.66–1.35)	0.7	
Multivariable HR (95% CI)^c^	Ref.	1.04 (0.75–1.45)	1.04 (0.73–1.48)	0.93 (0.62–1.39)	0.8	
**Vitamin D intake from non-dairy** (µg/day)^a^ **Women**	≤ 1.968	≤ 2.918	≤ 4.297	> 4.297		
Event N	50	56	68	117		
Crude HR (95% CI)	Ref.	1.12 (0.76–1.64)	1.34 (0.93–1.94)	**2.30 (1.65–3.20)**	**< 0.001**	
Basic-adj. HR (95% CI)^b^	Ref.	1.06 (0.72–1.57)	1.06 (0.72–1.56)	1.27 (0.87–1.84)	0.2	
Multivariable HR (95% CI)^c^	Ref.	1.02 (0.69–1.51)	0.97 (0.65–1.44)	1.08 (0.71–1.65)	0.7	0.1

^a^ Quartiles based on baseline dietary intake in non-cases

^b^ Adjusted for sex, age, country

^c^ Adjusted for sex, age, country, body mass index, education, physical activity, smoking level and intensity, alcohol intake, coffee consumption, diabetes, energy intake

We performed lagged analyses by excluding the first 5 years of follow-up to reduce the possibility of reverse causation. Associations for calcium were less pronounced, but a non-significant positive trend was still evident (*p* for trend = 0.058) (supplementary Table 6). In a further sensitivity analysis including only those PD cases which had been labelled as ‘definite’ or ‘very likely’ (*n* = 314) we observed a significant positive association for higher levels of cheese intake with PD risk (*p* for trend = 0.037) when adjusted for potential confounders (supplementary Table 7). Associations observed for calcium in the main analysis were deemed non-significant.

## Discussion

In this study, we found no notable evidence for an association of PD risk with the consumption of dairy products or dietary vitamin D intake in the large EPIC4ND sub-cohort. However, we found a positive association between dairy consumption and PD risk when solely examining the UK cohort. Additionally, a significant positive association between higher dietary calcium intakes and PD risk in men, potentially confined to dairy calcium, and in ever smokers was evident.

Several previous studies examined the risk of PD in relation to the consumption of milk and/or dairy products and/or to intakes of calcium and vitamin D. They mostly reported on a likely increased risk of PD with high milk consumption, potentially confined to men; however, results are conflicting for other dairy products. A sub-study in the Nurses´ Health Study (NHS) and Health Professionals Follow-up Study (HPFS) cohort in the USA observed an 80% increased PD risk for men but not for women comparing the highest with the lowest dairy intake category [22]. In further analysis, that same study found higher intakes of cream cheese, other cheese and sour cream to be significantly associated with an increased PD risk in men. In our study, we could not confirm these positive associations for dairy and individual dairy products with PD risk in men since associations from crude models were deemed non-significant after adjustment.

Another publication within the NHS and HPFS cohorts, including an additional follow-up time of up to 12 years, also found no significant association of dairy product consumption with the risk of developing PD overall, in men or women. However, an elevated PD risk was observed among individuals with high intakes of low-fat dairy foods as well as skim and low-fat milk [10]. Similarly, we found every increment of 100 g of semi-skimmed milk to be associated with a 5% increased risk of PD. In contrast to the study by Hughes et al., we did not find associations for skimmed milk. A study conducted among men with Japanese ancestry from the Honolulu Heart Program observed a significantly increased PD risk with higher milk intakes, which also remained after additional calcium intake adjustment [23]. Similarly, a sub-study within the Cancer Prevention Study II-Nutrition cohort in the US reported a significant positive association for dairy intake with PD risk in men, which was largely explained by milk consumption. No association was found for cheese, yogurt or ice cream intake, or when examining the relationship in women [24]. Likewise, our study did not find a positive association for dairy or individual products with PD risk in women. Within EPIC, one investigation in the EPIC Greece cohort reported an elevated PD risk for every additional standard deviation of dairy and milk consumption, but not for yogurt and cheese [12]. Similarly, we observed a risk-increasing association for every 200 g of dairy or milk consumption for participants from the UK, but these findings were not replicated in any other cohort of the present study.

A recent meta-analysis of prospective cohort studies from the USA, Sweden, Finland and Greece reported a significantly increased PD risk comparing the highest vs. the lowest category of intake of total dairy and milk in men and women combined, but not individually [25]. One Mendelian randomization study examined the association between genetically predicted dairy intake and PD risk using a genetic variant located in the lactase gene (rs4988235) that has been shown to be significantly associated with 0.2 more servings of dairy per day. The study reported a higher PD risk to be associated with higher genetically predicted dairy intake in men, but not in women [26]. 

Our investigations cannot confirm findings from previous studies concerning a positive association between milk and dairy products with PD risk in men; however, null associations found in our study for women were largely consistent with previous publications.

### Comparison to other studies – calcium and vitamin D

In order to investigate mechanisms responsible for the potential association between dairy product consumption and PD risk observed in a number of investigations, certain studies explored the relationship between specific nutrients found in dairy products, notably calcium and vitamin D, with PD risk [22–24, 27]. In most studies, the authors observed an increasing PD risk with higher intakes of calcium from dairy sources, whereas no significant association could be seen for calcium consumed from non-dairy sources [22–24]. Our study can confirm these findings, as we found a positive association of calcium intakes overall and from dairy with PD risk which is potentially confined to men. We observed an association with PD risk only for calcium from dairy foods, and no association was found for dairy overall in our study. Therefore, the suspected positive association between calcium and PD risk may only be evident for calcium as present in milk and dairy products. The association may not be apparent in dairy products as a whole food, as calcium is only one of many components of milk. A recent investigation on prediagnostic plasma calcium levels and PD risk within the EPIC4ND cohort did not observe any significant association between calcium and PD risk, providing no evidence to verify a role of circulating calcium in the pathogenesis of PD [28]. However, since blood calcium levels are not strongly affected by nutritional intake, but rather balanced through calcium homeostasis, the study does not allow to rule out a role of dietary calcium in the pathogenesis of PD.

As mentioned before, we observed a significant positive association between higher dietary calcium intakes and PD risk in men and in ever smokers. To the best of our knowledge, none of the previous investigations observed these interesting associations in confined subgroups, which merits further in depths validation in other cohorts. Additionally, potential for chance findings remains due to non-significant heterogeneity among subgroups.

Despite lower serum vitamin D levels observed in PD cases compared to controls [13], some of the aforementioned prospective investigations observed a positive association for vitamin D intake from dairy with PD risk [22, 24]; including ours. However, this association could not be seen for total dietary vitamin D intake, possibly due to a small proportion of total vitamin D intake that was derived from dairy in the study cohort. Two of the aforementioned studies did not find any association neither from overall vitamin D intake nor from intake from dairy products with PD risk [10, 12]. This suggests that vitamin D may not be related to the development of PD and that other mechanisms might therefore be responsible for the results in the aforementioned studies on serum vitamin D levels. One Mendelian randomization study explored the association between genetically decreased 25-hydroxyvitamin D concentrations with PD in individuals of European descent from 15 cohorts to investigate a potential causal relationship [29]. The authors concluded that there is a lack of definite evidence supporting a role of vitamin D in PD.

### Biological explanation

Some evidence suggests that environmental organochlorines and other pesticides contribute to the pathogenesis of PD [30, 31]. It has been shown that a significant proportion of organic environmental chemicals can bioconcentrate in milk and adipose tissue [32, 33]. Therefore, the accumulation of pesticides represents a plausible mechanism through which the association between dairy product consumption and PD risk in previous studies could be explained. In the present study, no evidence for an association between dairy intake and PD could be observed. One potential explanation for this is that, particularly when considering this plausible mechanism, the present cohort may have been exposed to different levels of pesticides present in the dairy products they consumed. However, it is important to consider that associations between dietary intake and disease risk in general may vary across different populations, suggesting that such associations detected in one population may not necessarily apply to others. Therefore, the observed relationship between dairy products and PD risk in previous studies may not necessarily exist in the present population. Lastly, we cannot rule out the presence of chance variation in our study.

### Strengths and limitations

Our present study demonstrates several strengths. Firstly, it was conducted within a large cohort comprising over 183,000 participants and 600 PD cases across 6 European countries. To the best of our knowledge, this study represents the first large-scale prospective cohort investigation across several European populations examining the association between intakes of dairy products, calcium and vitamin D with the risk of PD. In addition, study strengths include a large follow-up time covering a median period of over 17 years. Furthermore, its prospective study design mitigates the likelihood of reverse causation and recall bias, two limitations which are commonly observed in retrospective analyses of diet-disease relationships [10]. Compared to case-control studies, prospective cohort studies are anticipated to exhibit reduced susceptibility to selection bias [11].

However, certain limitations of the present study need to be considered. Dietary data of the current study was solely obtained from dietary assessment at baseline, assuming that dietary habits remain stable throughout follow-up. Consequently, the study design lacks the ability to accommodate changes in eating habits over the course of the study. This may become particularly relevant within the context of non-motor PD symptoms such as loss of the olfactory sense, depression or fatigue which may cause shifts in dietary patterns. Similarly, lifestyle data in the present study were only obtained from baseline. Therefore, potential alterations in these parameters over the course of the study were not considered in the current analysis. We did not have access to information on supplementary calcium and vitamin D intake, limiting the interpretation of the results to food-derived nutrient intakes. Additionally, we had no information on sun habits, as UV radiation from sun exposure is the major source of vitamin D. Furthermore, the distribution of dietary intakes encompasses a substantial number of outliers which could potentially distort the results. We cannot rule out presence of publication bias which may result in the omission of null results from publication in studies similar to those in existing literature. Lastly, the informative value of single nutritional risk factor analyses is somewhat limited as we are not evaluating a complete nutritional profile. For a given total energy intake, a higher or lower consumption of dairy products is supposedly compensated by lower or higher intakes of other energy-containing foods. In the present study, we adjusted for energy intake to account for this.

## Conclusions

Investigations into our well designed and large cohort study did not reveal convincing evidence for an association between dietary intakes of dairy products or vitamin D and the risk of developing PD. However, a positive association between dietary calcium intakes and PD risk in men and in participants ever having smoked was evident which warrants further validation within diverse populations.

## Electronic supplementary material

Below is the link to the electronic supplementary material.

Supplementary Material 1

## Data Availability

EPIC data and biospecimens are available for investigators who seek to answer important questions on health and disease in the context of research projects that are consistent with the legal and ethical standard practices of the International Agency for Research on Cancer (IARC), WHO, and the EPIC centres. The primary responsibility for accessing the data, obtained in the frame of the present publication, belongs to the EPIC centres that provided them. Access to EPIC data can be requested to the EPIC Steering Committee, as detailed in the EPIC-Europe Access Policy.

## Declarations

### Disclaimer

Where authors are identified as personnel of the International Agency for Research on Cancer / World Health Organisation, the authors alone are responsible for the view expressed in this article and they do not necessarily represent the decisions, policy or views of the International Agency for Research on Cancer / World Health Organization.
