# Supplementary material for: Associations of milk, dairy products, calcium and vitamin D intake with risk of developing Parkinson´s disease within the EPIC4ND cohort
Source: Eur J Epidemiol. 2024 Dec 3;39(11):1251–65. doi: 10.1007/s10654-024-01183-9 (PMC11646269; doi:10.1007/s10654-024-01183-9)
Supplement: Supplementary file 1 — Supplementary Material 1 [file 10654_2024_1183_MOESM1_ESM.pdf]

Supplementary Information

to the study *"Associations of Milk, Dairy Products, Calcium and Vitamin D Intake with Risk of Developing Parkinson´s Disease within the EPIC4ND Cohort"*

Mareike Gröninger, Jara Sabin, Rudolf Kaaks, Pilar Amiano, Dagfinn Aune, Natalia Cabrera Castro, Marcela Guevara, Johnni Hansen, Jan Homann, Giovanna Masala, Geneviève Nicolas, Susan Peters, Carlotta Sacerdote, Maria-Jose Sánchez, Maria Santucci De Magistris, Sabina Sieri, Roel Vermeulen, Yujia Zhao, Christina M. Lill, Verena Katzke*

*corresponding author

Verena A. Katzke

Division of Cancer Epidemiology, German Cancer Research Center (DKFZ), Im Neuenheimer Feld 581, 69120, Heidelberg, Germany.

v.katzke@dkfz-heidelberg.de

Content

Suppl. Table 1 Analysis of the association between **sociodemographic, anthropometric and lifestyle variables**, and incident Parkinson´s disease in the EPIC4ND cohort: sex- and age-adjusted Hazard Ratios (HRs) and 95% Confidence Intervals (CIs) obtained from Cox proportional hazards model. 3

Suppl. Table 2 Analysis of the association between units of dietary intake of **skimmed, semi-skimmed and whole milk**, and incident Parkinson´s disease in the EPIC4ND cohort: crude and multivariable-adjusted Hazard Ratios (HRs) and 95% Confidence Intervals (CIs) obtained from Cox proportional hazards model... 5

Suppl. Table 3 Analysis of the association between quartiles of dietary intake of total dairy, milk, yogurt, cheese, calcium and vitamin D, and incident Parkinson´s disease **stratified by smoking status** in the EPIC4ND cohort: crude and multivariable-adjusted Hazard Ratios (HRs) and 95% Confidence Intervals (CIs) obtained from Cox proportional hazards model. 6

Suppl. Table 4 Analysis of the association between quartiles of dietary intake of total dairy, milk, yogurt, cheese, calcium and vitamin D, and incident Parkinson´s disease **stratified by BMI** in the EPIC4ND cohort: crude and multivariable-adjusted Hazard Ratios (HRs) and 95% Confidence Intervals (CIs) obtained from Cox proportional hazards model. 9

Suppl. Table 5 Analysis of the association between units of dietary intake of total dairy, milk, yogurt, cheese, calcium and vitamin D, and incident Parkinson´s disease **by country** in the EPIC4ND cohort: crude and multivariable-adjusted Hazard Ratios (HRs) and 95% Confidence Intervals (CIs) obtained from Cox proportional hazards model... 12

Suppl. Table 6 Sensitivity analysis of the association between quartiles of dietary intake of total dairy, milk, yogurt, cheese, calcium and vitamin D, and incident Parkinson´s disease in the EPIC4ND cohort **excluding the first 5 years of follow-up**: crude and multivariable-adjusted Hazard Ratios (HRs) and 95% Confidence Intervals (CIs) obtained from Cox proportional hazards model. 14

Suppl. Table 7 Sensitivity analysis of the association between quartiles of dietary intake of total dairy, milk, yogurt, cheese, calcium and vitamin D, and incident Parkinson´s disease in the EPIC4ND cohort **including only definite and very likely PD cases**: crude and multivariable-adjusted Hazard Ratios (HRs) and 95% Confidence Intervals (CIs) obtained from Cox proportional hazards model................ 16

Suppl. Table 1 Analysis of the association between **sociodemographic, anthropometric and lifestyle variables**, and incident Parkinson´s disease in the EPIC4ND cohort: sex- and age-adjusted Hazard Ratios (HRs) and 95% Confidence Intervals (CIs) obtained from Cox proportional hazards model.

Each characteristic was examined in separate sex- and age-adjusted models. Women had a significantly lower risk of developing PD than men. Every 5-year increase in age was associated with a 66 % increased risk in PD. A higher level of school education was significantly associated with a lower PD risk compared to no school education at all. Coffee drinkers, compared to subjects who drank no coffee at all, exhibited a significantly lower risk of PD, and every increment of 150 ml of coffee per day was associated with a 7 % decreased risk. Every additional 10g of intake of alcohol per day was marginally significantly associated with a 6 % decreased PD risk. Individuals with diabetes mellitus showed a significantly elevated PD risk. Associations that were observed in crude models of BMI, physical activity or energy intake with PD risk were deemed non-significant after adjustment for sex and age.

Characteristic	Event N	HR [a]	95% CI [a]	p-value
**Sex** [b]				
Male	311	—	—	
Female	291	0.53	0.45, 0.62	**<0.001**
**Age at recruitment** [c]				
≤ 35	3	—	—	
> 35 - ≤ 40	3	0.54	0.11, 2.67	0.4
> 40 - ≤ 45	11	0.80	0.22, 2.87	0.7
> 45 - ≤ 50	54	2.98	0.93, 9.54	0.066
> 50 - ≤ 55	70	3.23	1.02, 10.3	**0.047**
> 55 - ≤ 60	110	6.22	1.97, 19.6	**0.002**
> 60 - ≤ 65	172	11.2	3.57, 35.0	**<0.001**
> 65 - ≤ 70	96	17.7	5.60, 55.8	**<0.001**
> 70 - ≤ 75	73	25.1	7.90, 79.5	**<0.001**
> 75	10	30.7	8.44, 111	**<0.001**
**Age at recruitment** (5 years more) [c]	602	1.66	1.58, 1.74	**<0.001**
**BMI** (kg/m^2)	602	1.00	0.98, 1.02	0.8
**BMI** (categories, kg/m^2)				
Low BMI (<18.5)	6	1.34	0.60, 3.02	0.5
Normal BMI (≥ 18.5 - ≤ 24.9)	224	—	—	
High BMI (>24.9)	372	1.01	0.85, 1.19	>0.9
**Highest school education**				
None	49	—	—	
Primary school completed	237	0.51	0.37, 0.70	**<0.001**
Technical/Professional school	142	0.53	0.38, 0.74	**<0.001**
Secondary school	63	0.47	0.32, 0.69	**<0.001**
Longer education	84	0.52	0.36, 0.74	**<0.001**
**Physical activity**				
Inactive	64	—	—	

Moderately inactive	182	0.99	0.74, 1.33	>0.9
Moderately active	259	0.94	0.71, 1.25	0.7
Active	32	0.77	0.50, 1.17	0.2
**Smoking status and intensity**				
Never	305	—	—	
Current, 1-15 cig/day	38	0.53	0.38, 0.75	**<0.001**
Current, 16-25 cig/day	17	0.48	0.30, 0.79	**0.004**
Current, 26+ cig/day	2	0.22	0.06, 0.89	**0.034**
Former, quit <= 10 years	50	0.71	0.52, 0.96	**0.025**
Former, quit 11-20 years	39	0.53	0.38, 0.74	**<0.001**
Former, quit 20+ years	96	0.91	0.72, 1.15	0.4
Current, pipe/cigar/occas	32	0.70	0.49, 1.02	0.062
**Alcohol consumption** (g/d)				
Non drinker	113	1.18	0.93, 1.50	0.2
>0-6(M)/>0-3(W)	173	—	—	
>6-12(M)/>3-12(W)	140	1.04	0.83, 1.30	0.7
>12-24	93	0.98	0.76, 1.26	0.9
>24-60	73	0.86	0.65, 1.14	0.3
>60	10	0.62	0.32, 1.17	0.14
**Alcohol consumption** (10 g/d more)	602	0.94	0.89, 1.00	**0.042**
**Coffee consumption** (150 ml/d more)	602	0.93	0.89, 0.97	**0.001**
**Coffee consumption** (categories)				
No coffee	68	—	—	
Up to 1 cup	156	0.63	0.47, 0.84	**0.002**
1-3 cups	207	0.66	0.50, 0.87	**0.003**
3-5 cups	126	0.55	0.41, 0.75	**<0.001**
More than 5 cups	45	0.53	0.36, 0.77	**0.001**
**Diabetes mellitus**				
No	376	—	—	
Yes	32	1.67	1.16, 2.40	**0.006**
**Energy intake** (500 kcal/d more)	602	1.05	0.98, 1.12	0.2
**Energy intake** (categories, kcal/d)				
≤ 1500	79	0.81	0.62, 1.05	0.11
> 1500 - ≤ 2000	196	—	—	
> 2000 - ≤ 2500	173	1.01	0.82, 1.25	>0.9
> 2500 - ≤ 3000	102	1.11	0.86, 1.41	0.4
> 3000	52	0.97	0.71, 1.34	0.9

[a] HR = Hazard Ratio, CI = Confidence Interval

[b] age-adjusted [c] sex-adjusted

Suppl. Table 2 Analysis of the association between units of dietary intake of **skimmed, semi-skimmed and whole milk**, and incident Parkinson´s disease in the EPIC4ND cohort: crude and multivariable-adjusted Hazard Ratios (HRs) and 95% Confidence Intervals (CIs) obtained from Cox proportional hazards model.

**Characteristic**	**HR**	**95% CI**
**Skimmed milk** (units of 100g)		
Crude	**1.09**	**1.04, 1.15**
Adjusted[a]	0.99	0.94, 1.05
**Semi-skimmed milk** (units of 100g)		
Crude	**1.11**	**1.07, 1.16**
Adjusted[a]	**1.05**	**1.00, 1.10**
**Whole milk** (units of 100g)		
Crude	1.02	0.96, 1.08
Adjusted[a]	0.95	0.89, 1.01

[a] Adjusted for sex, age, country, body mass index, education, physical activity, smoking history, alcohol intake, coffee consumption, diabetes, energy intake

Suppl. Table 3 Analysis of the association between quartiles of dietary intake of total dairy, milk, yogurt, cheese, calcium and vitamin D, and incident Parkinson´s disease **stratified by smoking status** in the EPIC4ND cohort: crude and multivariable-adjusted Hazard Ratios (HRs) and 95% Confidence Intervals (CIs) obtained from Cox proportional hazards model.

Variable	Quartile				*P* for trend	P_{het}
	1 (lowest)	2	3	4 (highest)		
**Total dairy** (g/day)[a]						
(men/women)	≤ 156.19/≤ 184.95	≤ 294.29/≤ 302.69	≤ 476.02/≤ 456.96	> 476.02/> 456.96		
**Never smokers**						
Event N	62	76	82	102		
Crude HR (95% CI)	Ref.	1.10 (0.79 – 1.54)	1.16 (0.83 – 1.61)	**1.41 (1.03 – 1.94)**	**0.027**	
Multivariable HR (95% CI)[b]	Ref.	0.98 (0.70 – 1.37)	0.82 (0.58 – 1.16)	0.97 (0.68 – 1.39)	0.7	
**Ever smokers**						
Event N	56	48	77	91		
Crude HR (95% CI)	Ref.	0.93 (0.63 – 1.37)	**1.56 (1.11 – 2.20)**	**1.89 (1.36 – 2.64)**	**< 0.001**	
Multivariable HR (95% CI)[b]	Ref.	0.79 (0.53 – 1.17)	0.89 (0.61 – 1.30)	1.15 (0.78 – 1.71)	0.3	0.4
**Milk Intake** (g/day)[a]						
(men/women)	≤ 36.21/≤ 56.40	≤ 164.29/≤ 163.91	≤ 312.76/≤ 296.22	> 312.76/> 296.22		
**Never smokers**						
Event N	63	58	97	104		
Crude HR (95% CI)	Ref.	0.84 (0.59 – 1.20)	1.32 (0.96 – 1.81)	**1.45 (1.06 – 1.99)**	**0.001**	
Multivariable HR (95% CI)[b]	Ref.	0.73 (0.51 – 1.05)	0.96 (0.68 – 1.35)	0.96 (0.67 – 1.37)	0.7	
**Ever smokers**						
Event N	55	58	71	88		
Crude HR (95% CI)	Ref.	1.14 (0.79 – 1.65)	**1.49 (1.05 – 2.12)**	**1.84 (1.32 – 2.58)**	**< 0.001**	
Multivariable HR (95% CI)[b]	Ref.	0.88 (0.60 – 1.28)	0.79 (0.53 – 1.16)	0.92 (0.62 – 1.35)	0.7	0.7

**Yogurt Intake** (g/day)[a]						
(men/women)	0/≤ 1.19	≤ 17.86/≤ 35.71	≤ 89.29/≤ 100.00	> 89.29/> 100.00		
**Never smokers**						
Event N	118	63	65	76		
Crude HR (95% CI)	Ref.	**0.65 (0.48 – 0.88)**	**0.57 (0.42 – 0.78)**	**0.61 (0.46 – 0.82)**	**< 0.001**	
Multivariable HR (95% CI)[b]	Ref.	0.98 (0.71 – 1.35)	**0.73 (0.53 – 1.00)**	**0.71 (0.52 – 0.98)**	**0.014**	
**Ever smokers**						
Event N	102	49	49	72		
Crude HR (95% CI)	Ref.	**0.68 (0.48 – 0.95)**	**0.62 (0.44 – 0.87)**	0.95 (0.70 – 1.28)	0.4	
Multivariable HR (95% CI)[b]	Ref.	1.27 (0.89 – 1.81)	0.98 (0.69 – 1.40)	**1.55 (1.12 – 2.16)**	**0.040**	0.2
**Cheese Intake** (g/day)[a]						
(men/women)	≤ 13.71/≤ 15.33	≤ 25.71/≤ 30.00	≤ 47.74/≤ 51.61	> 47.74/> 51.61		
**Never smokers**						
Event N	94	86	87	55		
Crude HR (95% CI)	Ref.	0.93 (0.69 – 1.24)	0.97 (0.73 – 1.30)	**0.64 (0.46 – 0.89)**	**0.023**	
Multivariable HR (95% CI)[b]	Ref.	1.14 (0.84 – 1.54)	1.35 (0.98 – 1.85)	1.04 (0.70 – 1.53)	0.4	
**Ever smokers**						
Event N	81	67	69	55		
Crude HR (95% CI)	Ref.	0.82 (0.59 – 1.13)	0.80 (0.58 – 1.10)	**0.61 (0.44 – 0.87)**	**0.007**	
Multivariable HR (95% CI)[b]	Ref.	0.90 (0.65 – 1.25)	1.20 (0.85 – 1.70)	1.24 (0.81 – 1.90)	0.2	0.2
**Calcium Intake** (mg/day)[a]						
(men/women)	≤ 734.70/≤ 728.75	≤ 970.56/≤ 939.80	≤ 1249.38/≤ 1189.82	> 1249.38/> 1189.82		
**Never smokers**						
Event N	73	76	84	89		
Crude HR (95% CI)	Ref.	1.04 (0.75 – 1.43)	1.15 (0.84 – 1.57)	1.21 (0.89 – 1.65)	0.2	
Multivariable HR (95% CI)[b]	Ref.	0.98 (0.71 – 1.37)	1.02 (0.72 – 1.45)	1.09 (0.74 – 1.62)	0.6	

**Ever smokers**						
Event N	51	60	75	86		
Crude HR (95% CI)	Ref.	1.19 (0.82 – 1.72)	**1.51 (1.06 – 2.15)**	**1.74 (1.23 – 2.45)**	**< 0.001**	
Multivariable HR (95% CI)[b]	Ref.	1.01 (0.69 – 1.49)	1.22 (0.82 – 1.81)	**1.64 (1.06 – 2.53)**	**0.014**	0.10
**Vitamin D Intake** (µg/day)[a]						
(men/women)	≤ 2.87/≤ 2.21	≤ 4.46/≤ 3.25	≤ 6.83/≤ 4.83	> 6.83/> 4.83		
**Never smokers**						
Event N	58	61	92	111		
Crude HR (95% CI)	Ref.	1.04 (0.73 – 1.49)	**1.48 (1.06 – 2.05)**	**1.83 (1.33 – 2.52)**	**< 0.001**	
Multivariable HR (95% CI)[b]	Ref.	1.02 (0.71 – 1.48)	1.25 (0.87 – 1.79)	1.23 (0.81 – 1.85)	0.2	
**Ever smokers**						
Event N	61	73	59	79		
Crude HR (95% CI)	Ref.	1.22 (0.87 – 1.71)	1.02 (0.72 – 1.47)	1.32 (0.94 – 1.84)	0.2	
Multivariable HR (95% CI)[b]	Ref.	1.11 (0.78 – 1.59)	0.83 (0.56 – 1.23)	0.94 (0.60 – 1.47)	0.5	0.052

[a] Quartiles based on baseline dietary intake among non-cases.

[b] Adjusted for sex, age, country, body mass index, education, physical activity, smoking history, alcohol intake, coffee consumption, diabetes, energy intake

Suppl. Table 4 Analysis of the association between quartiles of dietary intake of total dairy, milk, yogurt, cheese, calcium and vitamin D, and incident Parkinson´s disease **stratified by BMI** in the EPIC4ND cohort: crude and multivariable-adjusted Hazard Ratios (HRs) and 95% Confidence Intervals (CIs) obtained from Cox proportional hazards model.

Variable	Quartile				*P* for trend	P_{het}
	1 (lowest)	2	3	4 (highest)		
**Total dairy** (g/day)[a]						
(men/women)	≤ 156.19/≤ 184.95	≤ 294.29/≤ 302.69	≤ 476.02/≤ 456.96	> 476.02/> 456.96		
**Normal weight (BMI < 25)**						
Event N	49	45	59	82		
Crude HR (95% CI)	Ref.	0.90 (0.60 – 1.34)	1.14 (0.78 – 1.66)	**1.50 (1.05 – 2.13)**	**0.008**	
Multivariable HR (95% CI)[b]	Ref.	0.81 (0.54 – 1.23)	0.74 (0.50 – 1.12)	1.05 (0.70 – 1.58)	0.7	
**Overweight (BMI ≥ 25)**						
Event N	69	80	103	115		
Crude HR (95% CI)	Ref.	1.17 (0.85 – 1.61)	**1.56 (1.15 – 2.11)**	**1.82 (1.35 – 2.46)**	**< 0.001**	
Multivariable HR (95% CI)[b]	Ref.	0.97 (0.70 – 1.34)	0.94 (0.68 – 1.31)	1.07 (0.76 – 1.51)	0.7	0.4
**Milk Intake** (g/day)[a]						
(men/women)	≤ 36.21/≤ 56.40	≤ 164.29/≤ 163.91	≤ 312.76/≤ 296.22	> 312.76/> 296.22		
**Normal weight (BMI < 25)**						
Event N	51	44	59	81		
Crude HR (95% CI)	Ref.	0.87 (0.58 – 1.30)	1.18 (0.81 – 1.72)	**1.59 (1.12 – 2.25)**	**0.002**	
Multivariable HR (95% CI)[b]	Ref.	0.76 (0.50 – 1.15)	0.83 (0.55 – 1.25)	1.06 (0.71 – 1.59)	0.5	
**Overweight (BMI ≥ 25)**						
Event N	67	73	112	115		
Crude HR (95% CI)	Ref.	1.07 (0.77 – 1.50)	**1.62 (1.19 – 2.19)**	**1.73 (1.28 – 2.33)**	**< 0.001**	
Multivariable HR (95% CI)[b]	Ref.	0.83 (0.59 – 1.17)	0.92 (0.66 – 1.28)	0.88 (0.62 – 1.23)	0.7	0.5

**Yogurt Intake** (g/day)[a]						
(men/women)	0/≤ 1.19	≤ 17.86/≤ 35.71	≤ 89.29/≤ 100.00	> 89.29/> 100.00		
**Normal weight (BMI < 25)**						
Event N	90	30	47	68		
Crude HR (95% CI)	Ref.	**0.34 (0.23 – 0.52)**	**0.49 (0.35 – 0.70)**	**0.64 (0.47 – 0.88)**	**0.017**	
Multivariable HR (95% CI)[b]	Ref.	**0.60 (0.39 – 0.93)**	0.73 (0.50 – 1.05)	0.89 (0.63 – 1.26)	0.5	
**Overweight (BMI ≥ 25)**						
Event N	133	85	69	80		
Crude HR (95% CI)	Ref.	1.01 (0.77 – 1.33)	**0.71 (0.53 – 0.95)**	0.87 (0.66 – 1.14)	0.085	
Multivariable HR (95% CI)[b]	Ref.	**1.56 (1.17 – 2.08)**	0.94 (0.70 – 1.27)	1.12 (0.83 – 1.51)	> 0.9	> 0.9
**Cheese Intake** (g/day)[a]						
(men/women)	≤ 13.71/≤ 15.33	≤ 25.71/≤ 30.00	≤ 47.74/≤ 51.61	> 47.74/> 51.61		
**Normal weight (BMI < 25)**						
Event N	54	72	64	45		
Crude HR (95% CI)	Ref.	1.15 (0.80 – 1.63)	1.01 (0.70 – 1.45)	0.72 (0.49 – 1.08)	0.080	
Multivariable HR (95% CI)[b]	Ref.	1.39 (0.97 – 1.99)	1.45 (0.98 – 2.15)	1.37 (0.85 – 2.21)	0.14	
**Overweight (BMI ≥ 25)**						
Event N	123	87	92	65		
Crude HR (95% CI)	Ref.	0.80 (0.61 – 1.05)	0.85 (0.65 – 1.11)	**0.60 (0.44 – 0.81)**	**0.002**	
Multivariable HR (95% CI)[b]	Ref.	0.90 (0.68 – 1.19)	1.20 (0.90 – 1.61)	1.03 (0.72 – 1.47)	0.5	0.7
**Calcium Intake** (mg/day)[a]						
(men/women)	≤ 734.70/≤ 728.75	≤ 970.56/≤ 939.80	≤ 1249.38/≤ 1189.82	> 1249.38/> 1189.82		
**Normal weight (BMI < 25)**						
Event N	49	46	73	67		
Crude HR (95% CI)	Ref.	0.93 (0.62 – 1.38)	1.43 (0.99 – 2.05)	1.28 (0.89 – 1.86)	**0.047**	
Multivariable HR (95% CI)[b]	Ref.	0.86 (0.57 – 1.30)	1.29 (0.86 – 1.93)	1.37 (0.86 – 2.18)	0.063	

**Overweight (BMI ≥ 25)**						
Event N	75	91	91	110		
Crude HR (95% CI)	Ref.	1.23 (0.91 – 1.67)	1.27 (0.93 – 1.72)	**1.56 (1.16 – 2.09)**	**0.004**	
Multivariable HR (95% CI)[b]	Ref.	1.09 (0.80 – 1.50)	1.04 (0.74 – 1.45)	1.31 (0.90 – 1.89)	0.2	0.5
**Vitamin D Intake** (µg/day)[a]						
(men/women)	≤ 2.87/≤ 2.21	≤ 4.46/≤ 3.25	≤ 6.83/≤ 4.83	> 6.83/> 4.83		
**Normal weight (BMI < 25)**						
Event N	37	56	54	88		
Crude HR (95% CI)	Ref.	1.48 (0.98 – 2.24)	1.41 (0.92 – 2.13)	**2.17 (1.48 – 3.18)**	**< 0.001**	
Multivariable HR (95% CI)[b]	Ref.	1.51 (0.99 – 2.33)	1.30 (0.81 – 2.08)	1.62 (0.97 – 2.73)	0.14	
**Overweight (BMI ≥ 25)**						
Event N	83	79	102	103		
Crude HR (95% CI)	Ref.	0.97 (0.71 – 1.32)	1.25 (0.93 – 1.67)	1.31 (0.98 – 1.76)	**0.021**	
Multivariable HR (95% CI)[b]	Ref.	0.88 (0.64 – 1.21)	0.97 (0.70 – 1.34)	0.87 (0.60 – 1.26)	0.6	0.17

[a] Quartiles based on baseline dietary intake among non-cases.

[b] Adjusted for sex, age, country, body mass index, education, physical activity, smoking history, alcohol intake, coffee consumption, diabetes, energy intake

Suppl. Table 5 Analysis of the association between units of dietary intake of total dairy, milk, yogurt, cheese, calcium and vitamin D, and incident Parkinson´s disease **by country** in the EPIC4ND cohort: crude and multivariable-adjusted Hazard Ratios (HRs) and 95% Confidence Intervals (CIs) obtained from Cox proportional hazards model.

**Characteristic**	**Italy, N = 64**		**Spain, N = 96**		**UK, N = 187**		**The Netherlands, N = 13**		**Sweden, N = 194**		**Germany, N = 48**	
	**HR**	**95% CI**	**HR**	**95% CI**	**HR**	**95% CI**	**HR**	**95% CI**	**HR**	**95% CI**	**HR**	**95% CI**
**Dairy** (units of 200g)												
Crude	0.76	0.53, 1.07	0.90	0.71, 1.14	**1.30**	**1.12, 1.51**	0.94	0.60, 1.46	1.02	0.91, 1.13	0.94	0.70, 1.25
Adjusted[a]	0.82	0.56, 1.20	0.87	0.68, 1.11	**1.21**	**1.02, 1.43**	1.01	0.64, 1.59	1.06	0.94, 1.21	0.97	0.71, 1.33
**Milk** (units of 200g)												
Crude	0.71	0.47, 1.07	0.81	0.62, 1.06	**1.33**	**1.12, 1.57**	1.08	0.67, 1.75	1.04	0.93, 1.18	0.85	0.51, 1.42
Adjusted[a]	0.75	0.48, 1.17	0.76	0.58, 1.01	**1.20**	**1.00, 1.44**	1.17	0.70, 1.94	1.09	0.95, 1.25	0.88	0.53, 1.47
**Yogurt** (units of 150g)												
Crude	0.74	0.35, 1.58	1.22	0.76, 1.97	0.77	0.48, 1.22	0.37	0.07, 1.87	0.91	0.76, 1.09	0.99	0.64, 1.52
Adjusted[a]	0.88	0.43, 1.79	1.27	0.81, 2.01	1.00	0.64, 1.56	0.38	0.07, 2.03	0.99	0.82, 1.19	1.03	0.66, 1.59
**Cheese** (units of 30g)												

Crude	1.04	0.88, 1.23	1.11	0.97, 1.26	1.28	0.99, 1.66	1.14	0.62, 2.10	0.96	0.82, 1.13	0.79	0.50, 1.25
Adjusted[a]	1.09	0.90, 1.32	1.14	0.99, 1.30	1.26	0.97, 1.64	1.33	0.70, 2.52	0.93	0.77, 1.12	0.79	0.48, 1.29
**Calcium** (units of 200mg)												
Crude	0.96	0.84, 1.09	1.01	0.93, 1.10	**1.18**	**1.09, 1.29**	0.98	0.74, 1.32	1.02	0.95, 1.09	0.91	0.76, 1.09
Adjusted[a]	0.95	0.80, 1.15	0.99	0.89, 1.09	**1.17**	**1.03, 1.32**	1.08	0.79, 1.46	1.01	0.92, 1.12	0.94	0.76, 1.17
**Vitamin D** (units of 2μg)												
Crude	0.99	0.69, 1.41	1.01	0.88, 1.16	1.12	0.99, 1.26	0.92	0.35, 2.46	**1.09**	**1.00, 1.18**	0.91	0.68, 1.20
Adjusted[a]	1.07	0.73, 1.57	1.01	0.87, 1.17	1.00	0.85, 1.16	1.44	0.64, 3.23	0.99	0.88, 1.12	0.91	0.67, 1.24

[a] Adjusted for sex, age, body mass index, education, physical activity, smoking history, alcohol intake, coffee consumption, diabetes, energy intake

Suppl. Table 6 Sensitivity analysis of the association between quartiles of dietary intake of total dairy, milk, yogurt, cheese, calcium and vitamin D, and incident Parkinson´s disease in the EPIC4ND cohort **excluding the first 5 years of follow-up**: crude and multivariable-adjusted Hazard Ratios (HRs) and 95% Confidence Intervals (CIs) obtained from Cox proportional hazards model.

Variable	Quartile				P for trend
	1 (lowest)	2	3	4 (highest)	
**Total dairy** (g/day)[a] (men/women)	≤ 156.19/≤ 184.95	≤ 294.29/≤ 302.69	≤ 476.02/≤ 456.96	> 476.02/> 456.96	
Event N	89	101	117	143	
Crude HR (95% CI)	Ref.	1.13 (0.85 – 1.50)	1.31 (0.99 – 1.73)	**1.60 (1.23 – 2.08)**	**< 0.001**
Multivariable HR (95% CI)[b]	Ref.	0.98 (0.74 – 1.31)	0.87 (0.65 – 1.17)	1.10 (0.81 – 1.49)	0.6
**Milk Intake** (g/day)[a] (men/women)	≤ 36.21/≤ 56.40	≤ 164.29/≤ 163.91	≤ 312.76/≤ 296.22	> 312.76/> 296.22	
Event N	92	87	132	139	
Crude HR (95% CI)	Ref.	0.94 (0.70 – 1.26)	**1.42 (1.09 - 1.85)**	**1.51 (1.16 – 1.97)**	**< 0.001**
Multivariable HR (95% CI)[b]	Ref.	0.76 (0.57 – 1.03)	0.90 (0.67 – 1.20)	0.90 (0.66 – 1.21)	0.8
**Yogurt Intake** (g/day)[a] (men/women)	0/≤ 1.19	≤ 17.86/≤ 35.71	≤ 89.29/≤ 100.00	> 89.29/> 100.00	
Event N	168	78	92	112	
Crude HR (95% CI)	Ref.	**0.61 (0.46 – 0.79)**	**0.64 (0.49 – 0.82)**	**0.76 (0.60 – 0.96)**	**0.014**
Multivariable HR (95% CI)[b]	Ref.	1.04 (0.79 – 1.38)	0.92 (0.70 – 1.19)	1.03 (0.79 – 1.34)	> 0.9
**Cheese Intake** (g/day)[a] (men/women)	≤ 13.71/≤ 15.33	≤ 25.71/≤ 30.00	≤ 47.74/≤ 51.61	> 47.74/> 51.61	
Event N	132	119	116	83	
Crude HR (95% CI)	Ref.	0.90 (0.71 – 1.16)	0.88 (0.69 – 1.13)	**0.64 (0.48 – 0.84)**	**0.002**
Multivariable HR (95% CI)[b]	Ref.	1.11 (0.86 – 1.43)	**1.33 (1.02 – 1.74)**	1.19 (0.86 – 1.65)	0.11

**Calcium Intake** (mg/day)[a] (men/women)	≤ 734.70/≤ 728.75	≤ 970.56/≤ 939.80	≤ 1249.38/≤ 1189.82	> 1249.38/> 1189.82	
Event N	103	97	122	128	
Crude HR (95% CI)	Ref.	0.94 (0.71 – 1.24)	1.19 (0.92 – 1.55)	1.25 (0.96 – 1.62)	**0.031**
Multivariable HR (95% CI)[b]	Ref.	0.90 (0.67 – 1.19)	1.11 (0.83 – 1.49)	1.30 (0.94 – 1.81)	0.058
**Vitamin D Intake** (µg/day)[a] (men/women)	≤ 2.87/≤ 2.21	≤ 4.46/≤ 3.25	≤ 6.83/≤ 4.83	> 6.83/> 4.83	
Event N	83	97	124	146	
Crude HR (95% CI)	Ref.	1.17 (0.87 – 1.57)	**1.48 (1.12 – 1.95)**	**1.73 (1.32 – 2.27)**	**< 0.001**
Multivariable HR (95% CI)[b]	Ref.	1.15 (0.85 – 1.56)	1.28 (0.94 – 1.74)	1.31 (0.93 – 1.86)	0.11

[a] Quartiles based on baseline dietary intake among non-cases.

[b] Adjusted for sex, age, country, body mass index, education, physical activity, smoking level and intensity, alcohol intake, coffee consumption, diabetes, energy intake

Suppl. Table 7 Sensitivity analysis of the association between quartiles of dietary intake of total dairy, milk, yogurt, cheese, calcium and vitamin D, and incident Parkinson´s disease in the EPIC4ND cohort **including only definite and very likely PD cases**: crude and multivariable-adjusted Hazard Ratios (HRs) and 95% Confidence Intervals (CIs) obtained from Cox proportional hazards model.

Variable	Quartile				P for trend
	1 (lowest)	2	3	4 (highest)	
**Total dairy** (g/day)[a] (men/women)	≤ 156.19/≤ 184.95	≤ 294.29/≤ 302.69	≤ 476.02/≤ 456.96	> 476.02/> 456.96	
Event N	73	64	83	94	
Crude HR (95% CI)	Ref.	0.87 (0.62 – 1.22)	1.13 (0.83 – 1.55)	1.29 (0.95 – 1.75)	**0.039**
Multivariable HR (95% CI)[b]	Ref.	0.73 (0.52 – 1.03)	0.77 (0.55 – 1.08)	0.88 (0.62 – 1.25)	0.6
**Milk Intake** (g/day)[a] (men/women)	≤ 36.21/≤ 56.40	≤ 164.29/≤ 163.91	≤ 312.76/≤ 296.22	> 312.76/> 296.22	
Event N	67	67	89	91	
Crude HR (95% CI)	Ref.	1.00 (0.71 – 1.40)	1.32 (0.96 - 1.81)	1.36 (0.99 – 1.87)	**0.017**
Multivariable HR (95% CI)[b]	Ref.	0.78 (0.55 – 1.10)	0.81 (0.57 – 1.14)	0.81 (0.57 – 1.15)	0.3
**Yogurt Intake** (g/day)[a] (men/women)	0/≤ 1.19	≤ 17.86/≤ 35.71	≤ 89.29/≤ 100.00	> 89.29/> 100.00	
Event N	113	62	67	72	
Crude HR (95% CI)	Ref.	**0.72 (0.53 – 0.98)**	**0.69 (0.51 – 0.94)**	**0.73 (0.54 – 0.98)**	**0.024**
Multivariable HR (95% CI)[b]	Ref.	1.16 (0.84 – 1.62)	0.93 (0.67 – 1.28)	0.90 (0.65 – 1.26)	0.4
**Cheese Intake** (g/day)[a] (men/women)	≤ 13.71/≤ 15.33	≤ 25.71/≤ 30.00	≤ 47.74/≤ 51.61	> 47.74/> 51.61	
Event N	83	77	85	69	
Crude HR (95% CI)	Ref.	0.93 (0.68 – 1.27)	1.03 (0.76 – 1.39)	0.83 (0.61 – 1.15)	0.4
Multivariable HR (95% CI)[b]	Ref.	1.12 (0.82 – 1.54)	**1.51 (1.09 – 2.10)**	1.35 (0.92 – 1.98)	**0.037**

**Calcium Intake** (mg/day)[a] (men/women)	≤ 734.70/≤ 728.75	≤ 970.56/≤ 939.80	≤ 1249.38/≤ 1189.82	> 1249.38/> 1189.82	
Event N	76	66	76	96	
Crude HR (95% CI)	Ref.	0.87 (0.63 – 1.21)	1.00 (0.73 – 1.38)	1.27 (0.94 – 1.71)	0.073
Multivariable HR (95% CI)[b]	Ref.	0.82 (0.58 – 1.16)	0.93 (0.65 – 1.32)	1.26 (0.86 – 1.86)	0.2
**Vitamin D Intake** (µg/day)[a] (men/women)	≤ 2.87/≤ 2.21	≤ 4.46/≤ 3.25	≤ 6.83/≤ 4.83	> 6.83/> 4.83	
Event N	67	72	82	93	
Crude HR (95% CI)	Ref.	1.07 (0.77 – 1.50)	1.22 (0.88 – 1.68)	**1.37 (1.00 – 1.88)**	**0.032**
Multivariable HR (95% CI)[b]	Ref.	1.06 (0.75 – 1.49)	1.09 (0.76 – 1.57)	1.20 (0.80 – 1.81)	0.4

[a] Quartiles based on baseline dietary intake among non-cases.

[b] Adjusted for sex, age, country, body mass index, education, physical activity, smoking level and intensity, alcohol intake, coffee consumption, diabetes, energy intake
